# Higher water and nutrient use efficiencies in savanna than in rainforest lianas result in no difference in photosynthesis

**DOI:** 10.1093/treephys/tpab099

**Published:** 2021-07-26

**Authors:** Yun-Bing Zhang, Da Yang, Ke-Yan Zhang, Xiao-Long Bai, Yang-Si-Ding Wang, Huai-Dong Wu, Ling-Zi Ding, Yong-Jiang Zhang, Jiao-Lin Zhang

**Affiliations:** CAS Key Laboratory of Tropical Forest Ecology, Xishuangbanna Tropical Botanical Garden, Chinese Academy of Sciences, Menglun, Mengla, Yunnan 666303, China; Center of Plant Ecology, Core Botanical Gardens, Chinese Academy of Sciences, Menglun, Mengla, Yunnan 666303, China; University of Chinese Academy of Sciences, No. 19(A) Yuquan Road, Shijingshan District, Beijing 100049, China; CAS Key Laboratory of Tropical Forest Ecology, Xishuangbanna Tropical Botanical Garden, Chinese Academy of Sciences, Menglun, Mengla, Yunnan 666303, China; Center of Plant Ecology, Core Botanical Gardens, Chinese Academy of Sciences, Menglun, Mengla, Yunnan 666303, China; CAS Key Laboratory of Tropical Forest Ecology, Xishuangbanna Tropical Botanical Garden, Chinese Academy of Sciences, Menglun, Mengla, Yunnan 666303, China; Center of Plant Ecology, Core Botanical Gardens, Chinese Academy of Sciences, Menglun, Mengla, Yunnan 666303, China; University of Chinese Academy of Sciences, No. 19(A) Yuquan Road, Shijingshan District, Beijing 100049, China; CAS Key Laboratory of Tropical Forest Ecology, Xishuangbanna Tropical Botanical Garden, Chinese Academy of Sciences, Menglun, Mengla, Yunnan 666303, China; Center of Plant Ecology, Core Botanical Gardens, Chinese Academy of Sciences, Menglun, Mengla, Yunnan 666303, China; University of Chinese Academy of Sciences, No. 19(A) Yuquan Road, Shijingshan District, Beijing 100049, China; CAS Key Laboratory of Tropical Forest Ecology, Xishuangbanna Tropical Botanical Garden, Chinese Academy of Sciences, Menglun, Mengla, Yunnan 666303, China; Center of Plant Ecology, Core Botanical Gardens, Chinese Academy of Sciences, Menglun, Mengla, Yunnan 666303, China; University of Chinese Academy of Sciences, No. 19(A) Yuquan Road, Shijingshan District, Beijing 100049, China; CAS Key Laboratory of Tropical Forest Ecology, Xishuangbanna Tropical Botanical Garden, Chinese Academy of Sciences, Menglun, Mengla, Yunnan 666303, China; CAS Key Laboratory of Tropical Forest Ecology, Xishuangbanna Tropical Botanical Garden, Chinese Academy of Sciences, Menglun, Mengla, Yunnan 666303, China; School of Biology and Ecology, University of Maine, Orono, ME 04469, USA; CAS Key Laboratory of Tropical Forest Ecology, Xishuangbanna Tropical Botanical Garden, Chinese Academy of Sciences, Menglun, Mengla, Yunnan 666303, China; Center of Plant Ecology, Core Botanical Gardens, Chinese Academy of Sciences, Menglun, Mengla, Yunnan 666303, China; Yuanjiang Savanna Ecosystem Research Station, Xishuangbanna Tropical Botanical Garden, Chinese Academy of Sciences, Yuanjiang, Yunnan 653300, China

**Keywords:** acquisitive strategy, conservative strategy, dry habitat, functional traits, trait economics spectrum, woody climber

## Abstract

Differences in traits between lianas and trees in tropical forests have been studied extensively; however, few have compared the ecological strategies of lianas from different habitats. Here, we measured 25 leaf and stem traits concerning leaf anatomy, morphology, physiology and stem hydraulics for 17 liana species from a tropical seasonal rainforest and for 19 liana species from a valley savanna in south-west China. We found that savanna lianas had higher vessel density, wood density and lower hydraulically weighted vessel diameter and theoretical hydraulic conductivity than tropical seasonal rainforest lianas. Compared with tropical seasonal rainforest lianas, savanna lianas also showed higher leaf dry matter content, carbon isotope composition (δ^13^C), photosynthetic water use efficiency, ratio of nitrogen to phosphorus, photosynthetic phosphorus use efficiency and lower leaf size, stomatal conductance and nitrogen, phosphorus and potassium concentrations. Interestingly, no differences in light-saturated photosynthetic rate were found between savanna and tropical seasonal rainforest lianas either on mass or area basis. This is probably due to the higher water and nutrient use efficiencies of savanna lianas. A principal component analysis revealed that savanna and tropical seasonal rainforest lianas were significantly separated along the first axis, which was strongly associated with acquisitive or conservative resource use strategy. Leaf and stem functional traits were coordinated across lianas, but the coordination or trade-off was stronger in the savanna than in the tropical seasonal rainforest. In conclusion, a relatively conservative (slow) strategy concerning water and nutrient use may benefit the savanna lianas, while higher nutrient and water use efficiencies allow them to maintain similar photosynthesis as tropical seasonal rainforest species. Our results clearly showed divergences in functional traits between lianas from savanna and tropical seasonal rainforest, suggesting that enhanced water and nutrient use efficiencies might contribute to the distribution of lianas in savanna ecosystems.

## Introduction

Water availability is one of the main environmental factors limiting plant abundance and distribution (Woodward 1987). The projected increase in drought frequency and severity with climate change in many regions of the world poses a significant threat to the survival of plants (Case et al. 2019, Brodribb et al. 2020). Using data from 69 tropical forests worldwide with mean annual precipitation (MAP) >500 mm and seasonality of 3–4 months, Schnitzer (2005) found that liana abundance increases significantly with decreasing MAP and increasing seasonality. In addition, possibly associated with changes in precipitation patterns, there is accumulating evidence that liana abundance and biomass are rising (Phillips et al. 2002, Parolari et al. 2020, but see Smith et al. 2017). Therefore, investigating the variation in ecological strategy of lianas under different water availability scenarios has the potential to provide insights into understanding the role of lianas in forest structure, functioning and carbon sequestration (Gallagher and Leishman 2012, Schnitzer 2018, Estrada-Villegas et al. 2020).

Functional traits are defined as anatomical, morphological, physiological and phenological properties that influence plant growth, reproduction and survival, thereby fitness (Violle et al. 2007). Recent decades have seen the rise of applying functional traits to address the variation of plant ecological and life history strategies (Wright et al. 2007, Reich 2014). The ‘fast-slow plant economics spectrum’ (Reich 2014) predicts that different plant organs must be coordinated to converge in a unique ecological strategy continuum, which shifts from fast to slow resource acquisition and conservation due to evolutionary and biophysical constraints. Adaptation to contrasting habitats would lead to large functional trait variation (Reich 2014, Lohbeck et al. 2015, Medeiros et al. 2019). For instance, species with high resource acquisition-related traits are associated with resource-rich habitats (Asefa et al. 2017). By contrast, slow traits are advantageous for plants in low-resource settings due to the enhanced survival related to resource conservation. In addition, previous studies have found that plants adapt to various environmental conditions via multiple traits simultaneously (Carvajal et al. 2019, Medeiros et al. 2019). Therefore, habitat may act as an ecological filter to sieve species with different functional strategies, resulting in different habitats having biota with distinct ecological trait combinations (Lebrija-Trejos et al. 2010, Asefa et al. 2017). Most trait-based studies focus on trees. To date, we know little about how liana strategies vary among different habitats.

Previous studies on liana functional traits have predominantly concentrated in tropical forests (Zhu and Cao 2009, Chen et al. 2015, Schnitzer 2018, Werden et al. 2018), where liana richness and abundance are high. Lianas have thin, slender stems and are able to use other woody plants for mechanical supports (Darwin 1867, Schnitzer 2018). Lianas have a reduced requirement for mechanical support. Therefore, they can invest more energy or carbon in other life-history traits such as those related to high water transport and photosynthesis to aspects (Putz 1983, Jiménez-Castillo and Lusk 2013, Werden et al. 2018). As such, they usually possess acquisitive strategy traits, such as high specific leaf area, wide vessels, strong stomatal conductance, light-saturated photosynthetic rate and high hydraulic conductance, but are sensitive to drought-induced cavitation (Zhu and Cao 2009, Chen et al. 2015). Studies on liana functional traits outside tropical rainforests are rare (but see Jiménez-Castillo and Lusk 2013, Ganthaler et al. 2019). In particular, there are few studies focusing on the functional traits of savanna lianas. Zhang et al. (2016) found that a liana species possessed significantly higher stem- and leaf-specific hydraulic conductivity than a tree species within the same family in a Chinese valley savanna. In addition, there are few studies regarding the comparison of liana functional traits among different ecosystems. For example, Durigon et al. (2014) found that the frequency of growth forms and climbing mechanisms differed between subtropical and temperate areas in South America. Compared with temperate climbing plant species, tropical climbing plants had greater seed mass, leaf size (LS) and a relatively higher proportion of woody growth form (Gallagher et al. 2011). In a regional study comparing liana biodiversity and functional traits in four tropical forest types in India, Parthasarathy et al. (2015) found that leaf habit, climbing mechanism, flowering type and dispersal mode differed. In a global study of trait variation and evolution of climbing plants, Gallagher and Leishman (2012) found that climbers’ phylogenetic patterns differed among biogeographic regions and from other plant growth forms. All these studies have suggested that lianas from different habitats may exhibit different traits associated with different ecological strategies, but we still lack data on a comparison between savanna and tropical seasonal rainforest, which differ strikingly in precipitation regimes. Although a few easily measured liana functional traits were included in the above-mentioned comparative studies, relatively complete data on functional traits, including morphological, physiological and anatomical properties, are needed to better understand the ecological adaptation strategies of lianas to different habitats.

In the present study, we conducted a comparison of 25 stem and leaf traits for 36 liana species from two contrasting habitats, 17 from a tropical seasonal rainforest in Xishuangbanna and 19 from a valley savanna ecosystem in Yuanjiang, Yunnan Province, south-west China. Liana species are abundant in the tropical seasonal rainforest in Xishuangbanna (Liu et al. 2021). Compared with the tropical seasonal rainforest, the savanna possesses lower liana richness and abundance (Wu 1995, Jin and Ou 2000, Zhang et al. 2020). Our major objective was to evaluate how lianas differ in their ecological adaptation strategies when facing habitats with contrasting precipitation. Specifically, we attempted to answer the following three questions and test the corresponding hypotheses: (i) Do lianas from the savanna show more conservative ecological adaptation strategies compared to those from the tropical seasonal rainforest? Due to the limited water availability, we hypothesize that there exists a general ‘slow-fast’ (conservative-acquisitive) strategy across lianas, with savanna lianas exhibiting a relatively more conservative water use strategy than tropical seasonal rainforest lianas. (ii) Is there a difference in light-saturated photosynthetic rate between savanna and tropical seasonal rainforest lianas? We hypothesize that savanna lianas would have lower light-saturated photosynthetic rate than tropical seasonal rainforest lianas owing to the lower water availability, smaller xylem vessels and thus lower water transport (Pérez-Harguindeguy et al. 2013). (iii) Do savanna lianas show stronger linkages than tropical seasonal rainforest lianas among leaf and stem water-related traits? Because of strong environmental filtering associated with water availability in savanna ecosystems, we hypothesize that savanna lianas are able to regulate water transport and use water more efficiently than tropical seasonal rainforest lianas.

## Materials and methods

### Sites and species

This study was conducted in a tropical seasonal rainforest (hereafter ‘rainforest’) in Xishuangbanna (21°55′39″N, 101°15′55″E, elevation 570 m a.s.l.) and in a savanna ecosystem in Yuanjiang (23°27'56″N, 102°10'40″E, elevation 481 m a.s.l.), Yunnan Province, south-west China. These two sites are characterized by contrasting water conditions (Table 1). The MAP in Xishuangbanna is 1413 mm, whereas the MAP in Yuanjiang is only 733 mm. Both sites show a strong seasonality in precipitation, with a rainy season from May to October and with a dry season from November to next April. The aridity index (Nastos et al. 2013) of Yuanjiang savanna site (0.33) is much lower than that in the Xishuangbanna tropical rainforest site (0.96). Both Xishuangbanna and Yuanjiang have a hot climate, with a mean annual temperature of 22.7 and 24.7 °C, respectively. In the savanna site, soil water is abundant during the middle of the rainy season and the vapor pressure deficit (VPD) is 1.5–2.5 KPa in the morning, while VPD can reach higher than 3.5 KPa at noon due to high midday air temperatures of 28–35 °C. Despite higher concentrations of total and available soil nutrients, savanna lianas are able to use a small amount of nutrients due to the high proportion of sand in the soil- and water-deficit conditions. Yuanjiang savanna is characterized by typical rocky substrates (ca. 60–70% of rock outcrops) and very thin soil; the area lacks groundwater reserves, reducing plant water availability.

**Table 1:** The detailed description of climate and soil properties for tropical seasonal rainforest and savanna habitats.

Habitat type	Tropical seasonal rainforest	Savanna
Elevation (m a.s.l.)	580	481
Mean annual temperature (°C)	22.7	24.7
MAP (mm)	1447	733
Aridity index	0.96	0.33
Potential evaporation (mm)	1507	2220
pH	5.41	7.88
Organic matter (mg g^−1^)	46.91	87.73
Total N (mg g^−1^)	3.07	3.96
Total P (mg g^−1^)	0.69	1.30
Total K (mg g^−1^)	10.84	12.72
Available N (mg kg^−1^)	126.9	206.9
Available P (mg kg^−1^)	4.22	13.28
Available K (mg kg^−1^)	88.2	576.3

Aridity index was calculated following Nastos et al. (2013). Lower value of aridity index indicates the site is much drier. The duration of climate data for tropical seasonal rainforest is 1959–2018 and for savanna is 2012–17.

There is high liana richness and abundance in the Xishuangbanna tropical seasonal rainforest (Liu et al. 2021). Compared with rainforest, the richness and abundance of lianas are lower in the savanna (Wu 1995, Jin and Ou 2000). We chose 17 common liana species from the rainforest (Table S1 available as Supplementary data at *Tree Physiology* Online), among which *Byttneria integrifolia* Lace., *Combretum latifolium* Bl. and *Gnetum montanum* Markgr. are the most common liana species (Liu et al. 2021). We selected 19 liana species from the savanna site, representing ca. 85% of the liana flora in this site.

### Functional traits

We measured 25 functional traits characterizing water, nutrient and carbon economies for all selected 36 species from both sites, following a standardized protocol proposed by Pérez-Harguindeguy et al. (2013). The ecological significance of all traits measured is provided in Table 2. We collected data from three to five randomly selected individuals with a height around 5–10 m and a diameter of 2–5 cm per species. Sun-exposed branches with a diameter of ca. 1 cm were collected with a pole pruner. Branches were wrapped with moist paper tissues, put in plastic bags and shipped to the laboratory. These branches were rehydrated prior to further stem and leaf trait measurements. For compound leaf species, we used the leaflets instead. All traits were measured during the rainy season to avoid seasonal bias. Note that data on leaf anatomical, morphological, stomata and vein parameters of 17 liana species from the rainforest were collected from Ding et al. (2014), and data on leaf morphological, stomata, vein and stem traits of 19 savanna liana species were collected from Wu (2016), which were measured with the same methods. These two studies were conducted in two normal years, with precipitation of the rainforest site and that of the savanna site being 1679 and 792.5 mm (cf. Table 1), respectively.

**Table 2:** The ecological significance for each functional trait measured.

Trait	Code	Ecological significance	Unit
Leaf thickness	LT	Thicker leaves with higher LMA, longer leaf lifespan and lower relative growth rate (Wright et al. 2004, Westoby and Wright 2006)	μm
Palisade mesophyll thickness	PT	Palisade and spongy mesophylls are the main tissues for efficiently intercepting and transmitting light, thus optimizing photosynthesis (Terashima et al. 2011)	μm
Spongy mesophyll thickness	ST		μm
Stomatal density	SD	More stomata per area enables greater CO_2_ assimilation and promotes growth and competition (Tanaka and Shiraiwa 2009)	no. mm^−2^
Guard cell length	GCL	Larger guard cells and stomata result in large pores, enabling greater CO_2_ assimilation and promoting growth and competition (Hetherington and Woodward 2003)	μm
Leaf density	LD	Less dense leaves are associated with lower LMA and thus higher potential relative growth rate, and dense leaves with higher LMA, longer leaf lifespan and lower relative growth rates (Niinemets 2001, Westoby and Wright 2006)	kg m^−3^
Leaf mass per area	LMA	A lower LMA is associated with shorter leaf lifespan and higher resource acquisition capacity, indicating a fast-growth strategy (Wright et al. 2004)	g cm^−2^
Leaf size	LS	LS is thought to affect water loss. Smaller leaves have thinner boundaries, enabling leaves to keep cool especially when transpiration cooling is not possible during drought (Wright et al. 2017)	cm^2^
Leaf dry matter content	LDMC	Lower LDMC is related to lower LMA and thus higher potential relative growth rate. Lower LDMC may also be linked with drought tolerance in certain ecosystems (Niinemets 2001)	g g^−1^
Nitrogen concentration	N	Higher N concentrations per leaf area or mass are linked with more rapid photosynthetic rate per leaf area or mass, respectively (Wright et al. 2004)	mg g^−1^
Phosphorus concentration	P	Higher P concentrations per leaf area or mass are linked with more rapid photosynthetic rate per leaf area or mass, respectively (Wright et al. 2004)	mg g^−1^
Potassium concentration	K	K content was measured because it is involved in osmotic regulation in cells and is considered to be important for regulating stomatal opening (Benlloch-González et al. 2008)	mg g^−1^
N/P ratio	N/P	N/P is expected to detect the nature of nutrient limitation, with N/P <14 indicating N limitation, while N/P >16 P limitation (Koerselman and Meuleman 1996)	
Stable carbon isotope composition	δ^13^C	To estimate the efficiency of long-term water use in natural vegetations (Farquhar et al. 1989)	‰
Stomatal conductance	*g*_s_	Higher *g*_s_ leads to higher potential CO_2_ assimilation rate and thereby greater productivity and competition (Franks and Beerling 2009)	mol m^−2^ s^−1^
Area-based light-saturated photosynthetic rate	*A*_a_	Higher *A*_a_ relates to greater productivity and competition (Franks and Beerling 2009)	μmol m^−2^ s^−1^
Mass-based light-saturated photosynthetic rate	*A*_m_	Higher *A*_m_ relates to greater productivity and competition (Franks and Beerling 2009)	nmol^−2^ g^−1^ s^−1^
Photosynthetic nitrogen use efficiency	PNUE	It is inversely related to the leaf lifespan, positively related to photosynthesis (Poorter and Evans 1998)	μmol mol^−1^ s^−1^
Photosynthetic phosphorus use efficiency	PPUE	It is inversely related to the leaf lifespan, positively related to photosynthesis (Poorter and Evans 1998)	mmol mol^−1^ s^−1^
Photosynthetic water use efficiency	WUE_i_	Indicator of instantaneous water use efficiency; it is negatively related to the PNUE, PPUE (Santiago et al. 2004)	μmol mol^−1^
Leaf vein density	*D*_vein_	Higher vein densities would increase leaf hydraulic conductance and potentially photosynthetic rate and growth (Sack and Scoffoni 2013)	mm mm^−2^
Wood density	WD	WD is strongly related to the mechanical strength and capacity to prevent vessel implosion, water storage capacity and life history strategy (Hacke and Sperry 2001, Chave et al. 2009)	g cm^−3^
Vessel density	VD	VD is related to hydraulic transportation capacity; the higher the VD, the higher the water transport efficiency (Hacke and Sperry 2001, Chave et al*.* 2009)	no. mm^−2^
Hydraulically weighted vessel diameter	*D*_h_	Vessel diameter is related to hydraulic transport efficiency and is inversely related to the cavitation resistance (Hacke and Sperry 2001)	μm
Theoretical hydraulic conductivity	*K*_t_	Higher hydraulic conductance is related to higher stomatal conductance and higher photosynthetic carbon gain (Brodribb and Feild 2000, Santiago et al. 2004)	kg m^−1^ s^−1^ MPa^−1^

#### Leaf anatomy and morphology

We obtained leaf cross sections by freehand sectioning, avoiding midrib or large veins, and then we took pictures with a compound microscope (Leica Microsystems Ltd, Leica DM2500, Wetzlar, Germany). In total, 15–25 images were used to calculate the palisade mesophyll thickness (PT; μm), spongy mesophyll thickness (ST; μm) and leaf thickness (LT; μm) using ImageJ software (National Institutes of Health, Bethesda, MD, USA).

Leaf or leaflet size (LS; cm^2^) was determined by a flatbed scanner with 300-dpi resolution and we analyzed the scanned pictures by the ImageJ. We then put leaves with petioles removed into distilled water for more than 12 h and determined the saturated weight using a balance (0.0001 g; Mettler Toledo, AL204, Shanghai, China). Finally, leaves were oven-dried at 80 °C for at least 48 h to constant mass, then weighted. Leaf mass per area (LMA; g cm^−2^) was calculated as leaf dry mass divided by the fresh leaf area. Leaf dry matter content (LDMC; g g^−1^) was leaf dry mass divided by saturated weight. Leaf density (LD; kg m^−3^) was calculated as LMA/LT.

We utilized nail varnish impression method to prepare stomata slides. In the case of species that were too hairy, CH_3_COOH:H_2_O solution (1:1) was used to isolate the lower epidermis at 80 °C for 8–10 h. We measured guard cell length (GCL; μm) and stomatal density (SD; no. mm^−2^) from stomatal slides with the ImageJ software. The stomatal density was calculated as the number of stomata per unit area.

For leaf vein density, we sampled ca. 1 cm^2^ leaf segments avoiding the midrib and boiled them in 7% NaOH solution for 10–30 min. After bleaching the samples, we dyed them with 5% safranin solution. We took pictures using a compound microscope. At least 15–25 images were used for calculations. Vein density (*D*_vein_; mm mm^−2^) was calculated as the total vein length per leaf area.

#### Nutrient concentrations and carbon isotope composition

Fresh leaf samples with petioles removed were oven-dried at 80 °C for at least 48 h, then ground to fine powder and passed through a 60-mesh sieve. Nitrogen concentration (N; mg g^−1^) was measured by a Dumas-type combustion C-N elemental analyzer (Vario MAX CN, ElementarAnalysensysteme GmbH, Hanau, Germany). Phosphorus (P; mg g^−1^) and potassium (K; mg g^−1^) were measured with an inductively coupled plasma atomic-emission spectrometer (iCAP 7400, Thermo Fisher Scientific, Bremen, Germany). Stable carbon isotopic discrimination (δ^13^C; ‰) was measured using an isotope ratio mass spectrometer (IsoPrime100, Isoprime Ltd, Cheadle, Manchester, UK) against the Pee Dee Belemnite standard. We calculated δ^13^C as follows:

(1) \begin{equation*} {\delta}^{13}\mathrm{C}=\left[\frac{\left({R}_{\mathrm{sample}}\right)}{\left({R}_{\mathrm{standard}}\right)}-1\right]\times 1000, \end{equation*}

where *R*_sample_ and *R*_standard_ are the ratios of ^13^C/^12^C in the sample and the Pee Dee Belemnite standard, respectively.

#### Photosynthesis

We measured photosynthesis for sun-exposed fully expanded healthy leaves in vivo on intact branches in both habitats during the peak of the rainy season using a portable photosynthesis system (Li-6400, LiCor, Lincoln, Nebraska, USA). In the savanna, sun-exposed leaves are easily accessible for in vivo measurements due to the short stature of lianas and supporting trees. In the rainforest, we selected relatively short but mature individuals in relatively open areas. If the selected individual was tall and beyond the reach of the measurement, we carefully used a pruner pole to pull (not cut) an intact branch down until it was within reach and then measured the photosynthesis in vivo. We ensured that we did not damage the branch with the pruner pole while pulling. This is possible because lianas have thin, slender and flexible stems, which use other woody plants for mechanical support (Darwin 1867, Schnitzer 2018). We selected three to five individuals per species, and one leaf per individual, for photosynthetic gas exchange measurements. We selected the same individuals measured for nutrients, morphology and anatomy to determine photosynthesis. The measurements were conducted under a photosynthetic photon flux density of 1500 μmol m^−2^ s^−1^, ambient temperature (range of values between 25 and 30 °C), a CO_2_ concentration around 400 μmol mol^−1^ and relative humidity of 37–51% in the savanna and of 50–82% in the rainforest. Stomatal conductance (*g*_s_; mol m^−2^ s^−1^) and area-based light-saturated photosynthetic rate (*A*_a_; μmol m^−2^ s^−1^) were measured in situ between 9:00 and 11:00 a.m. Mass-based light-saturated photosynthetic rate (*A*_m_; nmol g^−1^ s^−1^) was calculated by dividing the area-based light-saturated photosynthetic rate by LMA (*A*_a_/LMA/10). Photosynthetic N use efficiency (PNUE; μmol mol^−1^ s^−1^) and photosynthetic P use efficiency (PPUE; mmol mol^−1^ s^−1^) were calculated as *A*_m_/N and *A*_m_/P, respectively. Intrinsic photosynthetic water use efficiency (WUE_i_; μmol mol^−1^) was calculated as *A*_a_/*g*_s_.

#### Wood traits

We debarked and removed the pith of stems, immersed stem samples in distilled water for 12 h until saturation, and the fresh volume of wood was determined using the water displacement method. All the samples were then oven-dried at 80 °C for 72 h and were weighed. Wood density (WD; g cm^−3^) was calculated as the ratio of wood dry mass to fresh volume. Other wood samples were subsequently fixed in formaldehyde acetic acid alcohol for further anatomical analyses.

We scraped and smoothed the 2–3 cm-length wood segments with a razor blade, then directly took pictures utilizing an automated digital microscope (ZEISS Smart zoom 5, Germany). PhotoShop CS5 (Adobe Systems, San Jose, CA, USA) was used to sharpen images for better distinguishing vessels from other tissues. For each individual, the wedge-shaped sectors under the field of view were selected to take pictures. At least 10–15 images from three individuals were used to calculate vessel diameter and vessel density (VD) for each species. Vessel density (no. mm^−2^) was calculated as the number of vessels in a unit cross section area. Each vessel was treated as an elliptical shape, and the major and minor axis dimensions of the vessels were then measured using the ImageJ to calculate the vessel diameter. Vessel diameter was calculated as follows:

(2) \begin{equation*} {D}_{\mathrm{i}}={\left(\frac{32{(ab)}^3}{a^2+{b}^2}\right)}^{\frac{1}{4}}, \end{equation*}

where *a* and *b* represent the radii of the major and minor axes of each vessel, respectively. The hydraulically weighted vessel diameter (*D*_h_; μm) was determined according to Poorter et al. (2010):

(3) \begin{equation*} {D}_{\mathrm{h}}={\left[\left(\frac{1}{n}\right)\sum \limits_{i=1}^n{D}_{\mathrm{i}}^4\right]}^{\frac{1}{4}}, \end{equation*}

According to Hagen-Poiseuille law (Tyree and Ewers 1991), the stem theoretical hydraulic conductivity (*K*_t_; kg m^−1^ s^−1^ MP_a_^−1^) was calculated as:

(4) \begin{equation*} {K}_{\mathrm{t}}=\left(\frac{\pi \rho}{128\eta A}\right)\sum \limits_{i=1}^n{D}_{\mathrm{i}}^4, \end{equation*}

where π is the circular constant of 3.14, ρ is the density of water (997.05 kg m^−3^ at 25 °C), and η is the viscosity of water (0.89 × 10^−9^ MPa s at 25 °C) and *A* is the area of images (Poorter et al. 2010).

### Statistical analyses

We first averaged all of the trait values for each species. The values of δ^13^C were converted from negative to positive by multiplying −1 to facilitate further analyses. All traits were log_10_-transformed to improve normality and homoscedasticity. The differences in functional traits between savanna and rainforest lianas were analyzed by independent samples *t*-test using the *t.test* function in the ‘stats’ package. During multiple comparisons, we used the method of Benjamini and Hochberg (1995) to adjust the *P*-values. Pearson’s correlations were used to quantify relationships among traits in two sites, which were calculated by the rcorr function in the ‘Hmisc’ package in R. To determine whether the trade-offs or coordination among traits differed in two sites, we performed standardized major axis analysis using the sma function of the ‘smatr’ package (Warton et al. 2012). We also conducted principal component analysis (PCA) using the rda function in the ‘vegan’ package (Oksanen et al. 2013) to verify whether savanna and rainforest lianas were positioned in different multivariate trait spaces. The rda function in the ‘vegan’ package performs a PCA when traits include no independent variables. The adonis function was used to perform a permutational analysis of variance (PERMANOVA) (Anderson 2001). This analysis was carried out to ensure whether species with different leaf habits and habitat types could be differentiated by a combination of measured leaf and stem traits (Euclidean distances, number of permuted data sets = 1,000,000). In the PERMANOVA analysis, we retained the additive model because the interaction between habitat type and leaf type was insignificant. Type II sums of squares were utilized in the PERMANOVA analysis. Given that evolutionary history may influence the traits at the species level, we first checked the phylogenetic signals for all traits. The phylogenetic signal represents a quantitative measure used to verify the degree to which phylogeny predicts the ecological similarity of species (Blomberg et al. 2003). By conducting phylogenetic ANOVA, phylogenetically independent contrasts (PICs) and phylogenetic PCA, we can assess the impact of phylogeny on trait differences and associations (Felsenstein 1985, de la Riva et al. 2016). The phylogenetic tree of lianas (Figure S1 available as Supplementary data at *Tree Physiology* Online) was retrieved based on a new tool developed by Jin and Qian (2019). The phylogenetic tree was reconstructed by the phylo.maker function in the ‘V.PhyloMaker’ package in R (Jin and Qian 2019). Phylogenetic ANOVA was used to test the differences in 25 functional traits between savanna and rainforest lianas using the aov.phylo function in the ‘geiger’ package (Pennell et al. 2014). The PIC was analyzed in the ‘apply’ function in the ‘ape’ package (Paradis et al. 2004). The phylogenetic PCA was conducted using the phyl.pca function in the ‘phytools’ package (Revell 2012). All analyses were carried out in R v.3.6.3 (R Core Team 2019).

## Results

### Differences in stem and leaf functional traits between rainforest and savanna lianas

Compared with rainforest lianas, savanna lianas had significantly higher VD, WD and lower hydraulically weighted vessel diameter (*D*_h_) and theoretical hydraulic conductivity (*K*_t_) (Table 3; *P* < 0.05). Moreover, savanna lianas showed significantly higher LDMC, stable carbon isotope composition (δ^13^C), photosynthetic water use efficiency (WUE_i_) and lower LS, and stomatal conductance (*g*_s_) than rainforest lianas (*P* < 0.05). Regarding nutrient concentrations, savanna lianas had significantly lower N, P and K concentrations (*P* < 0.05). Interestingly, the light-saturated photosynthetic rate, either on area or mass basis, was not significantly different between savanna and rainforest lianas (*P* > 0.05). Other leaf anatomical and morphological, stomatal and vein traits also did not differ between savanna and rainforest lianas (*P* > 0.05). The results of phylogenetic ANOVA showed similar patterns with the results of traditional independent samples *t*-test. The differences in PPUE and WD between savanna and rainforest lianas became marginally significant; *K* and *K*_t_ were not significantly different after considering the phylogenetic effect.

**Table 3:** Means ± SE of 25 functional traits for tropical seasonal rainforest and savanna liana species. The *t*, *P* and adjusted *P*-values of independent-samples *t*-test, the *F* and phylogenetic *P*-values of phylogenetic ANOVA were given.

Traits	Rainforest (*n* = 17)	Savanna (*n* = 19)	*t*	*P*	Adjusted *P*	*F*	Phylogenetic *P*
LT	212.65 ± 22.28	200.11 ± 15.77	0.37	0.717	0.932	0.134	0.784
PT	69.06 ± 6.75	68.84 ± 5.60	−0.04	0.965	1.000	0.002	0.980
ST	95.06 ± 10.17	89.47 ± 10.30	0.56	0.582	0.796	0.310	0.628
SD	322.82 ± 37.83	252.58 ± 20.67	1.06	0.297	0.455	1.120	0.431
GCL	22.00 ± 1.29	23.72 ± 1.07	−1.13	0.265	0.431	1.282	0.412
LD	301.88 ± 30.28	338.74 ± 35.10	−0.79	0.434	0.627	0.628	0.510
LMA	56.24 ± 3.29	62.32 ± 6.61	−0.23	0.821	0.977	0.049	0.922
LS	80.87 ± 10.52	26.03 ± 5.19	4.72	**<0.001**	**0.000**	22.285	**0.020**
LDMC	0.24 ± 0.02	0.34 ± 0.02	−3.61	**0.001**	**0.004**	13.054	**0.020**
N	26.88 ± 1.38	21.93 ± 1.36	2.65	**0.012**	**0.035**	7.035	**0.039**
P	2.05 ± 0.20	1.36 ± 0.11	3.01	**0.005**	**0.018**	9.061	**0.020**
K	15.13 ± 1.98	10.2 ± 1.37	2.11	**0.043**	0.085.	4.434	0.118
N/P	14.67 ± 1.16	17.15 ± 1.04	−1.77	0.085	0.158	3.150	0.255
δ^13^C	−30.43 ± 0.33	−27.36 ± 0.23	7.78	**<0.001**	**0.000**	60.539	**0.020**
*g*_s_	0.32 ± 0.03	0.21 ± 0.02	2.74	**0.010**	**0.035**	7.215	**0.039**
*A*_a_	11.39 ± 0.75	11.62 ± 0.94	0.04	0.969	1.000	0.002	0.980
*A*_m_	215.88 ± 21.74	216.84 ± 26.98	0.27	0.786	0.974	0.075	0.902
PNUE	112.12 ± 9.28	140.42 ± 16.69	−1.18	0.247	0.428	1.386	0.412
PPUE	3.47 ± 0.30	5.34 ± 0.71	−2.19	**0.036**	0.084.	4.792	0.059.
WUE_i_	39.29 ± 3.12	65.00 ± 4.83	−4.79	**<0.001**	**0.000**	22.979	**0.020**
*D*_vein_	7.40 ± 0.81	6.85 ± 0.56	0.16	0.875	0.989	0.025	0.922
WD	0.42 ± 0.02	0.53 ± 0.03	−2.51	**0.017**	**0.044**	6.297	**0.039**
*D*_h_	119.24 ± 15.99	59.42 ± 6.43	4.27	**<0.001**	**0.001**	45.922	**0.020**
VD	14.47 ± 1.54	63.63 ± 11.20	−6.78	**<0.001**	**0.000**	18.201	**0.020**
*K*_t_	186.76 ± 84.62	38.92 ± 6.88	2.11	**0.042**	0.085.	4.444	0.098

Significant differences are indicated in bold. See Table 2 for trait abbreviations.

### Associations among leaf and stem functional traits

**Figure 1.**
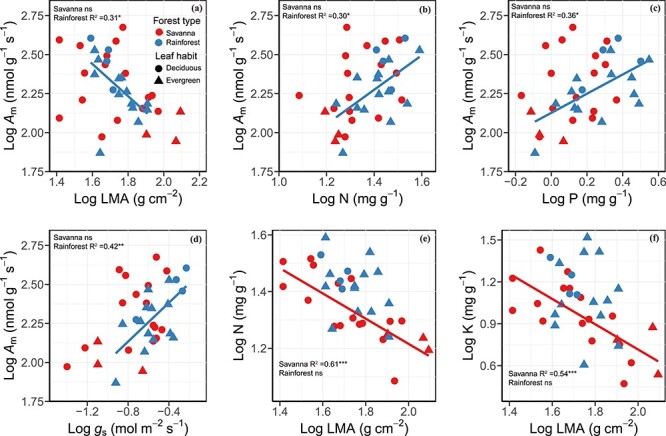
Log–log bivariate relationships between (a) mass-based light-saturated photosynthetic rate (*A*_m_) and LMA, (b) *A*_m_ and nitrogen concentration (N), (c) *A*_m_ and phosphorus concentration (P), (d) A_m_ and stomatal conductance (*g*_s_), (e) N and LMA and (f) potassium concentration (K) and LMA across liana species from savanna (red symbols and regression line; *n* = 19) and tropical seasonal rainforest (blue symbols and regression line; *n* = 17). Circle denotes deciduous lianas and triangle denotes evergreen lianas. Regression lines were given when bivariate correlations were significant. ns, *P* > 0.05; ^*^P < 0.05; ^*^^*^P < 0.01; ^*^^*^^*^P < 0.001.

The *A*_m_ was negatively associated with LMA and positively linked with N, P and *g*_s_ in rainforest lianas but not in savanna lianas (Figure 1). Leaf mass per area was negatively correlated with N and K in savanna lianas but not in rainforest lianas. Neither savanna nor rainforest lianas showed significant relationships between LMA and P (Tables S2 and S3 available as Supplementary data at *Tree Physiology* Online).

**Figure 2.**
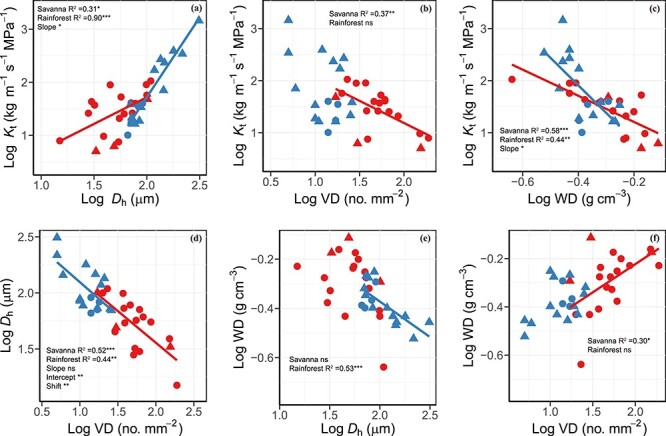
Log–log bivariate relationships between (a) theoretical hydraulic conductivity (*K*_t_) and hydraulically weighted vessel diameter (*d*_h_), (b) *K*_t_ and VD, (c) *K*_t_ and WD, (d) *d*_h_ and VD, (e) WD and *d*_h_ and (f) WD and VD across liana species from savanna (red symbols and regression line; *n* = 19) and tropical seasonal rainforest (blue symbols and regression line; *n* = 17). Circle denotes deciduous lianas and triangle denotes evergreen lianas. Regression lines were given when bivariate correlations were significant. Only when significant log–log bivariate relationships in two habitats were existent, the tests of the SMA regression slope, intercept and shift along the common slopes were conducted (see Table S4 available as Supplementary data at *Tree Physiology* Online). ns, *P* > 0.05; ^*^P < 0.05; ^*^^*^P < 0.01; ^*^^*^^*^P < 0.001.

For associations among stem hydraulic traits, *K*_t_ was positively related to *D*_h_, with savanna lianas having a significantly lower slope, and negatively related to WD, with savanna lianas having a significantly higher slope (Figure 2 and Table S4 available as Supplementary data at *Tree Physiology* Online). The *K*_t_ was negatively related to VD only in savanna lianas. The VD and *D*_h_ were negatively correlated in both sites, with savanna lianas having a significantly higher intercept and shift. The WD was negatively correlated with *D*_h_ only in rainforest lianas and was positively associated with VD only in savanna lianas.

**Figure 3.**
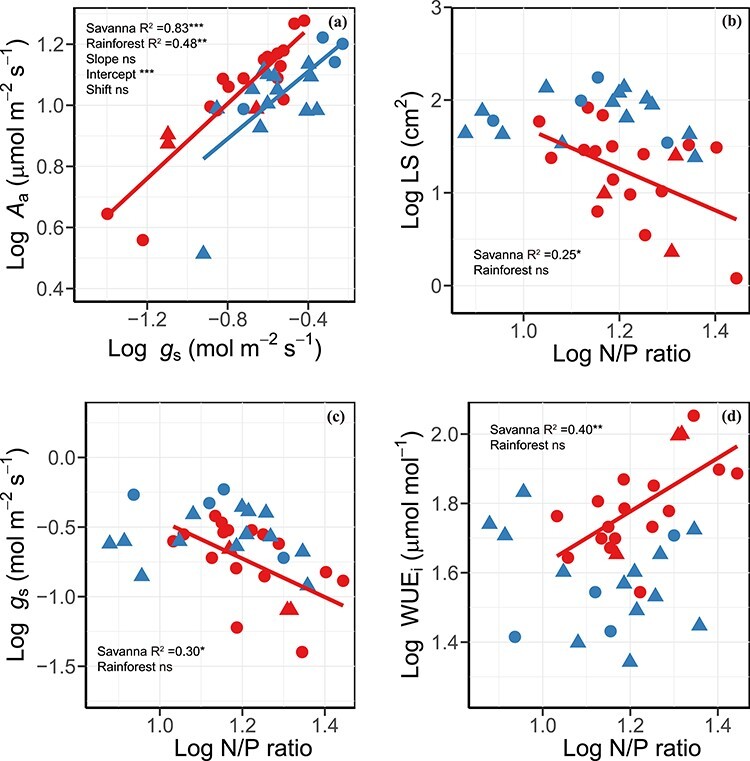
Log–log bivariate relationships between (a) area-based light-saturated photosynthetic rate (*A*_a_) and stomatal conductance (*g*_s_), (b) LS and N/P ratio, (c) *g*_s_ and nitrogen to phosphorus (N/P) ratio and (d) photosynthetic use efficiency (WUE_i_) and N/P ratio across liana species from savanna (red symbols and regression line; *n* = 19) and tropical seasonal rainforest (blue symbols and regression line; *n* = 17). Circle denotes deciduous lianas and triangle denotes evergreen lianas. See Table S4, available as Supplementary data at *Tree Physiology* Online, for the test of the SMA regression line between *A*_a_ and *g*_s_ in two habitats. ns, *P* > 0.05; ^*^P < 0.05; ^*^^*^P < 0.01; ^*^^*^^*^P < 0.001.

For associations among leaf water-related traits, *A*_a_ was positively related to *g*_s_ in two sites with a common slope, but savanna site had a significantly higher intercept than the rainforest site (Figure 3 and Table S4 available as Supplementary data at *Tree Physiology* Online). The N/P was negatively linked with LS and *g*_s_ and was positively associated with WUE_i_ only in savanna lianas.

**Figure 4.**
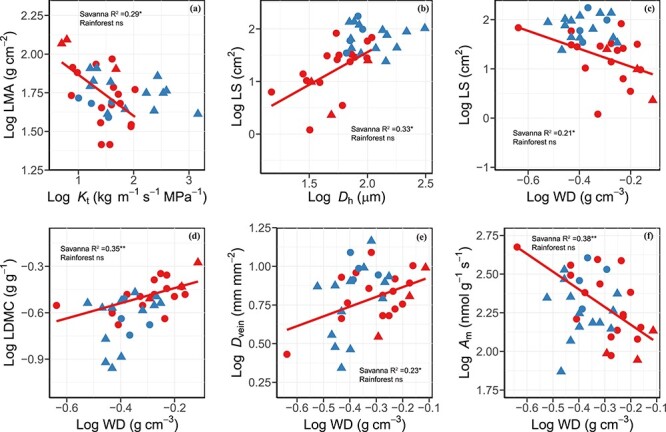
Log–log bivariate relationships between (a) LMA and theoretical hydraulic conductivity (*K*_t_), (b) LS and hydraulically weighted vessel diameter (*D*_h_), (c) LS and WD, (d) LDMC and WD, (e) vein density (*D*_vein_) and WD and (f) mass-based light-saturated photosynthetic rate (*A*_m_) and WD across liana species from savanna (red symbols and regression line; *n* = 19) and tropical seasonal rainforest (blue symbols and regression line; *n* = 17). Circle denotes deciduous lianas and triangle denotes evergreen lianas. Regression lines were given when bivariate correlations were significant. ns, *P* > 0.05; ^*^P < 0.05; ^*^^*^P < 0.01.

For associations between stem and leaf traits, LMA had a negative correlation with *K*_t_ in savanna lianas but not in rainforest lianas (Figure 4). Leaf size was positively related to *D*_h_ only in the savanna site. The WD was negatively correlated with LS and *A*_m_ and was positively correlated with LDMC and *D*_vein_ only in savanna lianas.

Our cross-species relationships between variables analyzed with Pearson’s and PIC correlations showed similar patterns (Tables S2 and S3 available as Supplementary data at *Tree Physiology* Online) owing to most traits having weak phylogenetic signals (Table S5 available as Supplementary data at *Tree Physiology* Online).

### Shift of lianas along the multivariate trait space

**Figure 5.**
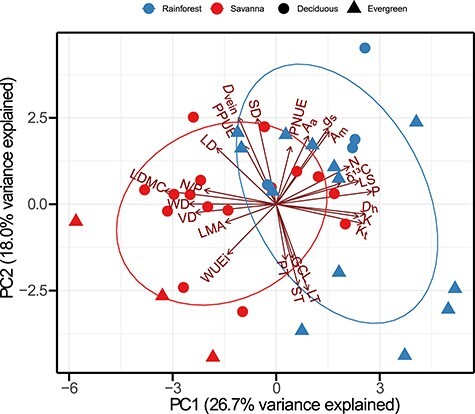
The biplot of the first two axes of the PCA for the 25 leaf and stem functional traits and the loadings of the 36 liana species from savanna (red) and tropical seasonal rainforest (blue). Circle denotes deciduous lianas and triangle denotes evergreen lianas. See text and Table 2 for trait abbreviations. All variables were log10-transfored before analysis. The unit of δ13C is (−‰).

Results of PCA based on 25 traits of 36 liana species from savanna and rainforest showed that the first and second components accounted for 26.7 and 18.0% of the total variance, respectively (Figure 5 and Table S6 available as Supplementary data at *Tree Physiology* Online). Phylogenetic PCA (Figure S2 available as Supplementary data at *Tree Physiology* Online) provided similar results with conventional PCA (Figure 5). The first axis was negatively correlated with traits representative of conservative resource use strategy (e.g., LDMC, WD, VD, N/P and δ^13^C). On the opposite were species with high trait values indicative of acquisitive resource use strategy (P, *D*_h_, *K*_t_, LS, K and N). Along the second axis, the positioning of lianas was attributed to *D*_vein_, LT, ST, SD, *g*_s_ and *A*_m_. Savanna lianas tended to be positioned at a relatively conservative end, and rainforest lianas were positioned at the acquisitive end. The multivariate trait space could be distinguished by the habitat type rather than leaf habit along the first axis (Table 4).

## Discussion

We found that savanna lianas exhibited a relatively conservative (slow) resource (water and nutrient) use strategy (e.g., higher LDMC, WD, VD, N/P ratio and δ^13^C; Figure 5 and Table 3). By contrast, rainforest lianas showed a relatively acquisitive (fast) strategy (higher P, hydraulically weighted vessel diameter, theoretical hydraulic conductivity, LS, N and K). These results agree with findings on trees along environmental gradients (de la Riva et al. 2016, Carvajal et al. 2019, Medeiros et al. 2019). Surprisingly, savanna and rainforest lianas did not differ in either area-based or mass-based light-saturated photosynthetic rate. Liana stem and leaf traits were strongly associated, suggesting a coordinated adaptation to environmental conditions between stems and leaves, which is in line with previous reports in trees (Reich 2014, Díaz et al. 2016, Carvajal et al. 2019). The coordination and/or trade-off concerning water conservation were stronger in savanna than in rainforest habitat, probably due to greater environmental constraints related to water availability in the savanna site. Previous studies have also shown that the coordination between leaf and stem traits tends to become stronger under harsh conditions (Dwyer and Laughlin 2017, Zeballos et al. 2017, Kawai and Okada 2019). Limited resources under harsh conditions may constrain the development of traits deviated from the coordination. In addition, we found that the associations among leaf or wood economics spectrum traits (Tables S2 and S3 available as Supplementary data at *Tree Physiology* Online) across lianas in two habitats are consistent with global patterns (Wright et al. 2004, Chave et al. 2009). However, we found some leaf economic spectrum traits decoupled in savanna or rainforest lianas. This is consistent with a previous report in which foliar P is uncorrelated with leaf economic spectrum axis in Bornean forest, which is characterized by strong edaphic resource gradients (Baltzer and Thomas 2010), indicating that soil conditions alter the relationships among leaf economic spectrum traits.

### Do savanna lianas exhibit relatively conservative water and nutrient use strategies compared to rainforest lianas?

In agreement with our first hypothesis, compared with rainforest lianas, we found that savanna lianas showed a relatively conservative water use strategy (higher LDMC, WD, VD, N/P ratio and δ^13^C). Notably, leaf phenology seems to contribute to the difference in functional traits between two sites. Most rainforest lianas (13 out of 17) are evergreen, while savanna lianas are mostly deciduous (16 out of 19). Because deciduous species generally have a more acquisitive resource use strategy (Givnish 2002, Fu et al. 2012, Zhang et al. 2013), the differences between savanna and rainforest lianas could be associated with leaf phenology. However, trait variation from different habitats might not be fully explained by leaf habits (Table 4 and Table S7 Supplementary data at *Tree Physiology* Online). Our results, therefore, suggest that lianas utilize different ecological strategies in different habitats. Consistent with previous results that lianas tend to have narrower vessels, lower hydraulic conductivity and higher cavitation resistance in dry forests than that in mesic forests (Carvalho et al. 2015, Carvalho et al. 2016), we found that savanna lianas have a higher WD, VD and lower hydraulically weighted vessel diameter and theoretical hydraulic conductivity (Table 3). Small vessels are potentially resistant to cavitation (Hacke and Sperry 2001, De Guzman et al. 2017). Furthermore, increasing evidence has demonstrated that WD is positively associated with cavitation resistance across species (Savi et al. 2018). Fu et al. (2012) also found that VD was positively related to cavitation resistance. It seems that higher WD, VD and lower hydraulically weighted vessel diameter and theoretical hydraulic conductivity could be essential for savanna lianas to cope with water deficits. In addition, some studies have reported that lianas have dimorphic vessels (Carlquist 1985, Zhu et al. 2017), which leads to their decoupled relationship between hydraulic efficiency and safety (Zhu et al. 2017). Therefore, vessel dimorphism could be incorporated into the future studies on ecological strategies of lianas in different habitats.

Small LS with a thinner boundary layer can speed the heat exchange with the surrounding environment, decreasing transpiration costs than larger leaves, thus reducing the risk of heat damage in the hot and dry environment (Wright et al. 2017). As for savanna lianas, smaller LS (Table 3) seems to be an advantage under dry and hot conditions, which is in line with the findings for lianas in semiarid habitats (Carvalho et al. 2016). Moreover, we found that the stomatal conductance of savanna lianas is much lower than that of rainforest lianas. Lower stomatal conductance could limit water loss, thereby avoiding or minimizing xylem dysfunction (Brodribb and Holbrook 2003) and favoring the survival of savanna lianas under relatively higher water deficits. Given that LDMC is related to the leaf modulus of elasticity or drought tolerance (Zimmermann 1978, Markesteijn et al. 2010), greater LDMC of savanna lianas presumably enhances their capability to survive under higher water deficits.

We found that savanna lianas tended to have lower N, P and K concentrations compared with rainforest lianas (Table 3). A similar pattern has been found in a comparison between savanna and forest trees in the Cerrado region of central Brazil (Hoffmann et al. 2005). Furthermore, we found that N/P ratio of savanna lianas (>16) was higher than their rainforest counterparts, indicating more P limitation on lianas in this dry and hot habitat (Koerselman and Meuleman 1996). Higher PPUE of savanna lianas may be an advantage over rainforest lianas in the P-limited habitat. We also found that savanna lianas exhibited higher intrinsic photosynthetic water use efficiency and δ^13^C values, a proxy of long-term water use efficiency (Farquhar et al. 1989), than rainforest lianas. Taken together, these results indicate that savanna lianas cope with water and nutrient limitations through enhanced water and nutrient use efficiencies.

### Is there a difference in light-saturated photosynthetic rate between savanna and rainforest lianas?

As leaf nutrient concentrations and water supply are two major determinants of light-saturated photosynthetic rate (Brodribb and Feild 2000, Wright et al. 2004, Zhang et al. 2015), significantly lower N, P, K and *g*_s_, hydraulically weighted vessel diameter and theoretical hydraulic conductivity in savanna lianas suggest potentially lower photosynthesis. Surprisingly, we did not find significant differences in both area- and mass-based light-saturated photosynthetic rate between savanna and rainforest lianas (Table 3), which is against our second hypothesis. This could be explained by higher PPUE and water use efficiency in savanna lianas, as mentioned above. More conservative water use and lower water supply in savanna lianas, indicated by narrower vessel diameter, lower hydraulic conductivity and higher WD, are compensated by increased intrinsic and long-term water use efficiency. This is also supported by the positive relationship between *A*_a_ and *g*_s_ in the two sites, with savanna lianas having a significantly higher intercept than rainforest lianas, indicating a higher water use efficiency in savanna lianas. This interesting pattern suggests that lianas can effectively regulate their resource use efficiency to maintain high photosynthesis under conditions with relatively low water and nutrient availability and low stem water transport.

Light-saturated photosynthetic rate of plants may be limited by structural constraints. For example, LMA is a key trait affecting photosynthesis (Wright et al. 2004). High LMA, thicker and denser leaves may increase the resistance of CO_2_ diffusion (Niinemets 2001), and low stomatal density could provide a deficient CO_2_ supply for assimilation (Tanaka and Shiraiwa 2009). Palisade and spongy mesophyll are the main tissues for efficiently intercepting and transmitting light, thus optimizing photosynthesis (Terashima et al. 2011). All these structural factors could influence light-saturated photosynthetic rate. However, we found no significant differences in leaf LMA, LT, LD, PT, ST, stomatal density and GCL between savanna and rainforest lianas. These trait syndromes may allow savanna lianas to maintain high carbon assimilation despite savanna site having lower moisture and nutrient availability than rainforest site. On the other hand, it suggests that the aggressive water use in rainforest lianas, which has also been reported in other studies (Chen et al. 2015, Campanello et al. 2016), probably consumes more water in terms of carbon assimilation (a low water use efficiency).

**Table 4:** *PERMANOVA* on Euclidean distance of 25 functional traits for 36 liana species from savanna (*n* = 19) and tropical seasonal rainforests (*n* = 17).

Source	d.f.	SS	MS	*F*	*R^2^*	*P*
Adonis (formula = species data ~ habitat type + leaf habit, data = group data, permutations = 1,000,000, method = ‘euclidean’)						
Habitat type	1	10.988	10.988	9.5723	0.213	0.001
Leaf habit	1	2.495	2.495	2.1737	0.049	0.058
Residual	33	37.879	1.147		0.737	
Total	35	51.362			1	
Adonis (formula = species data ~ leaf habit + habitat type, data = group data, permutations = 1,000,000, method = ‘euclidean’)						
Leaf habit	1	4.991	4.991	4.348	0.097	0.003
Habitat type	1	8.492	8.492	7.398	0.165	<0.001
Residual	33	37.879	1.147		0.738	
Total	35	51.362			1	

Note: d.f. is degrees of freedom, SS is sum of squares, MS is mean squares, *F* is the *F* value of the model, *R*^2^ is variance explained and *P* is the *P*-value of model.

### Do savanna lianas show stronger associations among water-related trait relationships than rainforest lianas?

We found tighter associations among water-related traits in savanna than in rainforest lianas, suggesting a stronger limitation of water supply to trait development in savanna lianas due to greater environmental constraints, supporting our third hypothesis. For stem-level traits, VD was negatively related to theoretical hydraulic conductivity only for savanna lianas. Since high VD is usually positively related to cavitation resistance (Fu et al. 2012), this relationship probably suggests that the trade-off between cavitation resistance and hydraulic efficiency is stronger in the savanna lianas due to the stronger environmental constraints. We also found that VD had a negative correlation with hydraulically weighted vessel diameter, with the savanna site having significantly higher intercept and shift, which is in accordance with a previous study (Preston et al. 2006). It may be very useful to allow a broad range of vessel traits at a given WD (Preston et al. 2006), especially in the dry and hot environment. Wood density was positively associated with VD only for savanna lianas, indicating that cavitation resistance might be more important in savanna lianas. For leaf-level traits, we found that N/P ratio of savanna lianas was positively correlated with photosynthetic water use efficiency and was negatively related to *g*_s_, which is in line with a finding from tropical trees (Cernusak et al. 2010). These strategies may be advantageous for savanna lianas to cope with water deficits.

Strong leaf and stem coordination related to water conservation was found in lianas in the savanna site but not in the rainforest. First, LS was positively associated with hydraulically weighted vessel diameter and was negatively correlated with WD only in savanna lianas, which is in line with previous studies on trees (Pickup et al. 2005, Kawai and Okada 2019), presumably indicating the balance between transpiration and water transport as well as the facilitation of high water transport in leaf expansion. Second, LDMC was positively linked with WD only in savanna lianas, indicating that there probably exists a strong coordination of drought tolerance between leaf and stem organs. Third, vein density was positively associated with WD for savanna lianas. Vein density is related to leaf mechanical strength, and WD is related to stem mechanical support (Roth-Nebelsick et al. 2001, Onoda et al. 2010); therefore, strong linkage between vein density and WD presumably suggests the coordination of mechanical support between leaves and stems. Fourth, WD was negatively associated with *A*_m_ only for savanna lianas, probably because low water transport associated with high WD constrains photosynthesis. Together, all of the above-mentioned associations in savanna lianas suggest strong linkages among traits related to conservative water use, either at stem-level, leaf-level or between stem and leaf. Many studies have also acknowledged that plants respond to changes in environmental conditions via trait coordination (Fu et al. 2012, Jager et al. 2015, Medeiros et al. 2019). However, some previous studies have shown that leaf and stem wood traits are decoupled in trees (e.g., Baraloto et al. 2010).

Most patterns revealed by traditional analyses were similar to those from phylogenetic ANOVA and phylogenetic PCA. We found that some cross-species correlations did not exist after taking phylogeny into account (e.g., N and P with *A*_m_ in the rainforest; LMA and *K*_t_, LS with *D*_h_ and WD in the savanna), indicating that the relationships are malleable or plastic. Although the main objective of this study was to test whether the species mean is different between two habitat types for each trait, intraspecific variation of these traits could also be studied to quantify the effect of spatial heterogeneity in water availability on functional traits. In addition, other abiotic and/or biotic factors other than precipitation regimes between two study sites, such as the geology and soils, may also play a role in the variation of functional traits. For instance, some studies have suggested that nutrient stress would alter xylem structure, reducing the vessel diameter, even in the absence of water stress (Beikircher et al. 2019, Cary et al. 2020). Hence, future studies should incorporate factors, such as soil texture, geology and competition from neighbors, for a better understanding of the differentiation in liana ecological strategies in different habitats.

## Conclusions

Our results clearly show that lianas in the dry habitat employ a relatively conservative (slow) resource use strategy compared to those from the wet habitat. Possessing slow/conservative traits in water-limited or low-resource environments would enhance plant survival (Reich 2014, Carvajal et al. 2019). Interestingly, conservative water and nutrient use of savanna lianas did not result in lower photosynthetic carbon assimilation, mainly due to increased water and nutrient use efficiencies through modification of leaf morphology and anatomy. Our results, focusing on a number of lianas and using a series of stem and leaf functional traits, revealed that the ecological strategies of lianas differed in habitats with contrasting water availability. Enhanced water and nutrient use efficiencies might have contributed to the ecological success of lianas in dry and hot habitats like savanna ecosystems.

## Supplementary Material

Supporting_Information-2021-04-14_zjl_revised_tpab099
