# Supplementary material for: Higher water and nutrient use efficiencies in savanna than in rainforest lianas result in no difference in photosynthesis
Source: Tree Physiol. 2021 Jul 26;42(1):145–59. doi: 10.1093/treephys/tpab099 (PMC8755031; doi:10.1093/treephys/tpab099)
Supplement: Supporting_Information-2021-04-14_zjl_revised_tpab099 [file supporting_information-2021-04-14_zjl_revised_tpab099.docx]

**Supplementary Data**

**Table S1.** Liana species with details of family and leaf habits in tropical seasonal rainforest and savanna habitats.

| Species | Family | Leaf habit | Habitat |
| --- | --- | --- | --- |
| *Byttneria* *integrifolia* Lace. | Sterculiaceae | Deciduous | Rainforest |
| *Caesalpinia* *cucullata* Roxb. | Fabaceae | Evergreen | Rainforest |
| *Combretum* *latifolium* Bl. | Combretaceae | Evergreen | Rainforest |
| *Combretum* *griffithii* var. *yunnanense* (Exell) Turland & C. Chen | Combretaceae | Deciduous | Rainforest |
| *Congea* *tomentosa* Roxb. | Verbenaceae | Deciduous | Rainforest |
| *Dalbergia* *rimosa* Roxb. | Fabaceae | Deciduous | Rainforest |
| *Fissistigma* *acuminatissimum* Merr. | Annonaceae | Evergreen | Rainforest |
| *Gnetum* *montanum* Markgr. | Gnetaceae | Evergreen | Rainforest |
| *Iodes* *cirrhosa* Turcz. | Icacinaceae | Evergreen | Rainforest |
| *Mucuna* *hainanensis* Hayata. | Fabaceae | Evergreen | Rainforest |
| *Smilax* *corbularia* Kunth. | Smilacaceae | Evergreen | Rainforest |
| *Stixis* *suaveolens* (Roxb.) Pierre | Capparaceae | Evergreen | Rainforest |
| *Tetrastigma* *cruciatum* Craib et Gagnep. | Vitaceae | Evergreen | Rainforest |
| *Tetrastigma* *obovatum* (Laws.) Gagnep. | Vitaceae | Evergreen | Rainforest |
| *Tetrastigma* *planicaule* (Hook.) Gagnep. | Vitaceae | Evergreen | Rainforest |
| *Uncaria* *laevigata* Wal. ex G. Don. | Rubiaceae | Evergreen | Rainforest |
| *Ventilago* *calyculata* var. *trichoclada* Y. L. Chen et P. K. Chou. | Rhamnaceae | Evergreen | Rainforest |
| *Abrus* *precatorius* L. | Fabaceae | Deciduous | Savanna |
| *Abrus* *pulchellus* Wall. ex Thwaites. | Fabaceae | Deciduous | Savanna |
| *Argyreia* *osyrensis* var. *cinerea* Hand.-Mazz. | Convolvulaceae | Deciduous | Savanna |
| *Bauhinia* *calciphila* D. X. Zhang & T. C. Chen. | Fabaceae | Deciduous | Savanna |
| *Bauhinia* *championii* (Benth.) Benth. | Fabaceae | Deciduous | Savanna |
| *Bridelia* *stipularis* (L.) Bl. | Phyllanthaceae | Deciduous | Savanna |
| *Cajanus* *mollis* (Benth.) van der Maesen. | Fabaceae | Deciduous | Savanna |
| *Capparis* *cantoniensis* Lour. | Capparaceae | Deciduous | Savanna |
| *Cissus* *repanda* Vahl. | Vitaceae | Deciduous | Savanna |
| *Cryptolepis* *buchananii* Roem. et Schult. | Apocynaceae | Deciduous | Savanna |
| *Cyclea* *wattii* Diels. | Menispermaceae | Deciduous | Savanna |
| *Dregea* *volubilis* (L. f.) Benth. ex Hook. f. | Apocynaceae | Deciduous | Savanna |
| *Gymnema* *sylvestre* (Retz.) Schult. | Apocynaceae | Deciduous | Savanna |
| *Jasminum* *seguinii* Lévl. | Oleaceae | Evergreen | Savanna |
| *Myriopteron* *extensum* (Wight et Arnott) K. Schum. | Apocynaceae | Deciduous | Savanna |
| *Passiflora* *henryi* Hemsl. | Passifloraceae | Deciduous | Savanna |
| *Phyllanthus* *reticulatus* Poir. | Phyllanthaceae | Deciduous | Savanna |
| *Pterolobium* *macropterum* Kurz. | Fabaceae | Evergreen | Savanna |
| *Secamone* *sinica* Hand.-Mazz. | Apocynaceae | Deciduous | Savanna |
| *Smilax* *china* L. | Smilacaceae | Evergreen | Savanna |

Nomenclature follows Flora of China (http://www.iplant.cn/foc/).

**Table S2.** Coefficients (*r*) of Pearson’s correlation (lower off-diagonal) and PIC correlation (upper off-diagonal) for 25 traits across17 tropical seasonal rainforest liana species.

|  | LT | PT | ST | SD | GCL | LD | LMA | LS | LDMC | N | P | K | N/P | δ^13^C | *g*_s_ | *A*_a_ | *A*_m_ | PNUE | PPUE | WUE_i_ | *D*_vein_ | WD | *D*_h_ | VD | *K*_t_ |
| --- | --- | --- | --- | --- | --- | --- | --- | --- | --- | --- | --- | --- | --- | --- | --- | --- | --- | --- | --- | --- | --- | --- | --- | --- | --- |
| LT |  | 0.41 | **0.61** | -0.39 | -0.10 | **-0.51** | 0.23 | **-0.51** | -0.12 | -0.29 | 0.25 | **0.46** | **-0.48** | -0.13 | -0.40 | -0.21 | -0.35 | -0.17 | **-0.45** | **0.54** | 0.03 | -0.03 | -0.04 | 0.17 | -0.14 |
| PT | **0.71** |  | 0.28 | 0.00 | 0.18 | -0.33 | -0.01 | -0.41 | -0.03 | -0.17 | 0.07 | 0.00 | -0.24 | 0.25 | -0.10 | 0.08 | -0.01 | 0.15 | 0.04 | 0.22 | 0.03 | 0.23 | -0.14 | 0.37 | -0.17 |
| ST | **0.82** | **0.60** |  | -0.32 | 0.04 | -0.24 | 0.33 | -0.23 | 0.12 | -0.14 | 0.15 | 0.04 | -0.19 | -0.38 | **-0.58** | **-0.58** | **-0.55** | **-0.57** | **-0.63** | **0.52** | -0.03 | 0.00 | -0.12 | 0.09 | -0.19 |
| SD | **-0.62** | -0.15 | **-0.53** |  | **-0.49** | **0.57** | 0.33 | 0.02 | **0.56** | -0.37 | -0.36 | -0.30 | 0.15 | 0.03 | 0.20 | 0.02 | -0.13 | 0.03 | 0.16 | -0.25 | 0.27 | **0.71** | **-0.54** | **0.50** | **-0.52** |
| GCL | 0.37 | 0.21 | 0.37 | **-0.70** |  | **-0.52** | **-0.64** | 0.16 | **-0.66** | 0.28 | 0.30 | -0.14 | -0.14 | -0.14 | **0.43** | **0.50** | **0.65** | **0.55** | 0.35 | -0.25 | **-0.53** | **-0.55** | 0.40 | **-0.56** | 0.30 |
| LD | **-0.84** | **-0.63** | **-0.65** | **0.65** | **-0.42** |  | **0.67** | 0.39 | **0.72** | -0.30 | **-0.65** | **-0.51** | **0.59** | 0.16 | -0.20 | -0.21 | **-0.43** | -0.37 | 0.10 | 0.05 | 0.05 | 0.17 | -0.13 | 0.27 | -0.11 |
| LMA | 0.09 | 0.00 | 0.15 | 0.18 | -0.16 | **0.46** |  | -0.01 | **0.81** | **-0.57** | **-0.49** | -0.28 | 0.27 | 0.06 | **-0.66** | **-0.54** | **-0.87** | **-0.68** | -0.40 | **0.46** | 0.12 | 0.25 | -0.22 | **0.45** | -0.27 |
| LS | -0.41 | -0.25 | -0.38 | 0.37 | -0.16 | **0.49** | 0.22 |  | -0.20 | 0.24 | -0.01 | -0.21 | 0.11 | 0.06 | 0.34 | 0.36 | 0.29 | 0.16 | 0.28 | -0.31 | -0.14 | **-0.47** | **0.43** | -0.31 | 0.35 |
| LDMC | **-0.59** | -0.40 | -0.25 | **0.57** | **-0.47** | **0.74** | 0.41 | -0.02 |  | **-0.58** | **-0.64** | **-0.48** | **0.46** | 0.16 | **-0.53** | **-0.61** | **-0.79** | **-0.61** | -0.26 | 0.17 | 0.21 | **0.50** | **-0.46** | **0.48** | **-0.47** |
| N | -0.27 | -0.03 | -0.24 | 0.25 | -0.18 | 0.01 | -0.41 | 0.38 | -0.22 |  | **0.58** | 0.39 | -0.09 | -0.17 | 0.10 | 0.13 | **0.43** | 0.00 | -0.06 | 0.03 | 0.20 | -0.19 | 0.30 | -0.19 | **0.55** |
| P | 0.37 | **0.43** | 0.15 | -0.06 | 0.05 | **-0.50** | -0.32 | 0.24 | **-0.70** | **0.57** |  | **0.68** | **-0.82** | -0.24 | 0.19 | 0.09 | 0.31 | 0.11 | **-0.47** | -0.13 | 0.38 | -0.02 | -0.03 | -0.02 | 0.06 |
| K | **0.48** | 0.21 | 0.23 | -0.31 | 0.08 | **-0.51** | -0.15 | 0.10 | **-0.70** | 0.27 | **0.65** |  | **-0.55** | -0.17 | 0.01 | 0.07 | 0.15 | 0.04 | -0.35 | 0.25 | **0.46** | -0.01 | -0.23 | 0.31 | -0.11 |
| N/P | **-0.61** | **-0.54** | -0.32 | 0.23 | -0.18 | **0.61** | 0.14 | -0.05 | **0.72** | -0.07 | **-0.86** | **-0.62** |  | 0.19 | -0.30 | -0.14 | -0.19 | -0.24 | 0.42 | 0.24 | -0.37 | -0.14 | 0.17 | -0.07 | 0.20 |
| δ^13^C | -0.38 | -0.34 | -0.31 | -0.07 | -0.10 | 0.28 | -0.11 | 0.05 | 0.29 | -0.24 | -0.41 | -0.32 | 0.35 |  | -0.05 | 0.04 | -0.04 | 0.09 | 0.24 | -0.26 | 0.14 | -0.12 | 0.28 | 0.12 | 0.20 |
| *g*_s_ | **-0.58** | -0.24 | **-0.71** | **0.60** | -0.21 | **0.44** | -0.15 | **0.58** | 0.08 | 0.18 | 0.22 | -0.16 | -0.16 | 0.11 |  | **0.81** | **0.86** | **0.89** | **0.63** | **-0.75** | -0.07 | -0.12 | 0.05 | -0.31 | -0.02 |
| *A*_a_ | -0.13 | 0.20 | **-0.42** | **0.50** | -0.22 | 0.11 | -0.02 | **0.54** | -0.29 | 0.39 | **0.51** | 0.15 | -0.38 | -0.37 | **0.69** |  | **0.87** | **0.92** | **0.77** | -0.37 | -0.26 | -0.30 | 0.30 | -0.32 | 0.22 |
| *A*_m_ | -0.16 | 0.17 | **-0.43** | 0.31 | -0.09 | -0.16 | **-0.56** | 0.33 | **-0.46** | **0.55** | **0.60** | 0.20 | -0.39 | -0.25 | **0.65** | **0.84** |  | **0.89** | **0.68** | **-0.51** | -0.25 | -0.32 | 0.30 | **-0.44** | 0.30 |
| PNUE | -0.04 | 0.21 | -0.38 | 0.23 | -0.01 | -0.20 | **-0.43** | 0.17 | **-0.42** | 0.06 | 0.38 | 0.08 | **-0.42** | -0.16 | **0.67** | **0.77** | **0.87** |  | **0.76** | **-0.55** | -0.29 | -0.23 | 0.18 | -0.35 | 0.07 |
| PPUE | **-0.58** | -0.29 | **-0.65** | **0.43** | -0.17 | 0.37 | -0.28 | 0.11 | 0.25 | -0.01 | **-0.42** | **-0.49** | **0.51** | 0.16 | **0.50** | 0.39 | **0.48** | **0.57** |  | -0.32 | **-0.48** | -0.29 | 0.33 | -0.33 | 0.25 |
| WUE_i_ | **0.66** | **0.55** | **0.53** | -0.30 | 0.06 | **-0.49** | 0.18 | -0.23 | **-0.43** | 0.16 | 0.25 | 0.39 | -0.21 | **-0.55** | **-0.63** | 0.12 | 0.01 | -0.09 | -0.27 |  | -0.07 | -0.02 | -0.02 | 0.31 | 0.06 |
| *D*_vein_ | **-0.75** | -0.34 | **-0.58** | **0.86** | **-0.70** | **0.71** | 0.07 | 0.40 | **0.65** | 0.31 | -0.09 | -0.35 | 0.30 | 0.26 | **0.61** | 0.31 | 0.21 | 0.07 | 0.35 | **-0.52** |  | **0.52** | **-0.49** | **0.58** | -0.33 |
| WD | -0.22 | 0.00 | -0.25 | **0.68** | **-0.55** | 0.31 | 0.22 | 0.06 | 0.34 | 0.01 | -0.01 | 0.14 | 0.02 | -0.22 | 0.26 | 0.21 | 0.06 | 0.06 | 0.08 | -0.13 | **0.44** |  | **-0.67** | **0.52** | **-0.50** |
| *D*_h_ | 0.23 | 0.07 | 0.14 | **-0.61** | **0.54** | -0.39 | -0.32 | -0.02 | **-0.49** | 0.14 | 0.09 | -0.12 | -0.02 | 0.13 | -0.19 | -0.06 | 0.13 | 0.07 | 0.05 | 0.20 | **-0.53** | **-0.73** |  | **-0.72** | **0.92** |
| VD | 0.17 | 0.35 | 0.06 | 0.34 | -0.23 | 0.05 | 0.38 | 0.14 | -0.02 | 0.07 | 0.36 | **0.46** | -0.39 | -0.26 | 0.08 | 0.26 | 0.00 | -0.04 | -0.39 | 0.17 | 0.18 | **0.45** | **-0.66** |  | **-0.61** |
| *K*_t_ | 0.35 | 0.24 | 0.21 | **-0.59** | **0.52** | **-0.46** | -0.26 | 0.06 | **-0.61** | 0.26 | 0.31 | 0.06 | -0.21 | 0.03 | -0.19 | 0.04 | 0.18 | 0.06 | -0.13 | 0.31 | **-0.54** | **-0.66** | **0.95** | **-0.42** |  |

Note: See Table 2 for trait abbreviations. Green font: *p* **<** 0.1; bold font: *p* **<** 0.05; red font: *p* **<** 0.01.The unit of δ^13^C is (–‰).

**Table S3.** Coefficients (*r*) of Pearson’s correlation (lower off-diagonal) and PIC correlation (upper off-diagonal) for 25 traits across19 savanna liana species.

|  | LT | PT | ST | SD | GCL | LD | LMA | LS | LDMC | N | P | K | N/P | δ^13^C | *g*_s_ | *A*_a_ | *A*_m_ | PNUE | PPUE | WUE_i_ | *D*_vein_ | WD | *D*_h_ | VD | *K*_t_ |
| --- | --- | --- | --- | --- | --- | --- | --- | --- | --- | --- | --- | --- | --- | --- | --- | --- | --- | --- | --- | --- | --- | --- | --- | --- | --- |
| LT |  | **0.8** | **0.92** | **0.5** | -0.35 | **-0.44** | 0.24 | -0.08 | -0.09 | -0.23 | -0.05 | -0.15 | -0.29 | -0.09 | 0.17 | 0.07 | -0.31 | -0.09 | -0.21 | -0.22 | **-0.4** | 0.1 | -0.01 | 0.27 | -0.04 |
| PT | **0.84** |  | **0.54** | **0.55** | -0.33 | -0.1 | **0.44** | 0.02 | 0.04 | -0.37 | -0.11 | -0.39 | -0.31 | -0.17 | 0.25 | 0.17 | -0.25 | -0.07 | -0.16 | -0.14 | -0.16 | 0.11 | -0.03 | 0.22 | 0.1 |
| ST | **0.94** | **0.63** |  | **0.45** | -0.35 | **-0.44** | 0.16 | -0.23 | -0.06 | -0.17 | -0.12 | -0.06 | -0.13 | -0.07 | 0.09 | 0 | -0.37 | -0.15 | -0.2 | -0.19 | -0.39 | 0.14 | -0.05 | 0.35 | -0.13 |
| SD | 0.24 | 0.33 | 0.19 |  | **-0.77** | 0.23 | **0.45** | -0.32 | 0.31 | **-0.6** | **-0.42** | **-0.53** | -0.17 | -0.29 | 0.21 | 0.15 | **-0.46** | -0.07 | -0.15 | -0.09 | 0.35 | **0.42** | 0.11 | **0.55** | 0.05 |
| GCL | -0.05 | -0.12 | -0.05 | **-0.47** |  | -0.04 | -0.3 | **0.47** | -0.27 | 0.36 | 0.32 | 0.3 | 0.02 | 0.23 | 0.21 | 0.15 | **0.47** | 0.25 | 0.25 | -0.2 | **-0.5** | **-0.44** | -0.1 | -0.36 | 0.05 |
| LD | -0.34 | -0.18 | -0.33 | 0.2 | 0.25 |  | 0.37 | -0.13 | 0.37 | -0.22 | -0.19 | -0.32 | 0.07 | -0.31 | 0.24 | 0.22 | -0.21 | -0.23 | -0.1 | 0.05 | **0.55** | 0.35 | 0.16 | 0.18 | 0.19 |
| LMA | **0.4** | **0.53** | 0.33 | 0.35 | 0.12 | 0.37 |  | -0.34 | **0.82** | **-0.71** | **-0.46** | **-0.75** | -0.14 | **-0.56** | 0.05 | -0.07 | **-0.5** | -0.2 | -0.24 | 0 | **0.47** | **0.63** | -0.2 | **0.47** | -0.38 |
| LS | 0.23 | 0.2 | 0.11 | -0.13 | **0.57** | -0.05 | -0.02 |  | **-0.61** | 0.31 | 0.37 | 0.2 | -0.12 | -0.21 | **0.48** | **0.67** | **0.77** | **0.64** | **0.45** | 0.18 | -0.37 | -0.36 | **0.43** | **-0.76** | 0.26 |
| LDMC | -0.22 | 0.03 | -0.24 | 0.03 | 0.05 | **0.43** | **0.58** | **-0.44** |  | **-0.65** | **-0.53** | **-0.63** | 0.02 | -0.25 | -0.33 | **-0.45** | **-0.66** | -0.4 | -0.33 | 0.09 | **0.57** | **0.72** | **-0.43** | **0.61** | **-0.56** |
| N | -0.27 | **-0.41** | -0.19 | -0.19 | -0.24 | -0.23 | **-0.78** | -0.12 | **-0.63** |  | **0.85** | **0.7** | -0.16 | 0.22 | 0.02 | 0.11 | **0.44** | -0.13 | -0.13 | -0.02 | **-0.43** | **-0.41** | 0.21 | **-0.54** | 0.17 |
| P | -0.06 | -0.2 | -0.07 | -0.08 | 0.12 | 0 | **-0.41** | 0.26 | **-0.6** | **0.72** |  | **0.58** | **-0.64** | 0.08 | 0.13 | 0.11 | 0.27 | -0.21 | **-0.41** | -0.25 | **-0.4** | -0.24 | 0.34 | **-0.49** | 0.1 |
| K | -0.08 | -0.27 | -0.05 | -0.24 | -0.03 | -0.27 | **-0.73** | 0.32 | **-0.76** | **0.7** | **0.62** |  | -0.03 | **0.51** | -0.2 | -0.09 | 0.19 | -0.12 | -0.08 | 0.04 | **-0.44** | **-0.52** | 0.35 | **-0.56** | 0.39 |
| N/P | -0.19 | -0.14 | -0.1 | -0.09 | **-0.43** | -0.25 | -0.24 | **-0.5** | 0.2 | 0.02 | **-0.68** | -0.16 |  | 0.17 | -0.18 | 0.03 | 0.21 | 0.3 | **0.67** | **0.52** | 0.13 | -0.25 | -0.29 | -0.03 | 0.17 |
| δ^13^C | -0.16 | -0.15 | -0.15 | -0.11 | -0.03 | -0.29 | **-0.64** | 0.01 | -0.24 | 0.36 | 0.11 | **0.59** | 0.23 |  | **-0.43** | **-0.53** | -0.11 | -0.23 | -0.06 | -0.17 | **-0.42** | -0.36 | -0.25 | 0.1 | 0.22 |
| *g*_s_ | 0.25 | 0.19 | 0.23 | **0.55** | 0.16 | 0.2 | 0.23 | 0.27 | -0.38 | 0 | 0.38 | -0.07 | **-0.55** | -0.28 |  | **0.86** | **0.44** | **0.48** | 0.33 | **-0.41** | -0.1 | -0.24 | 0.29 | -0.13 | 0.31 |
| *A*_a_ | 0.17 | 0.12 | 0.15 | **0.6** | 0.04 | 0.2 | 0.17 | 0.29 | **-0.43** | 0.06 | 0.3 | -0.07 | -0.37 | -0.39 | **0.91** |  | **0.62** | **0.62** | **0.52** | 0.06 | -0.05 | -0.31 | 0.38 | **-0.41** | 0.39 |
| *A*_m_ | -0.27 | -0.34 | -0.26 | 0.16 | -0.12 | **-0.42** | **-0.43** | 0.13 | **-0.56** | **0.41** | 0.29 | 0.21 | 0.02 | 0.02 | **0.42** | **0.55** |  | **0.81** | **0.73** | 0.13 | -0.27 | **-0.67** | 0.21 | **-0.66** | 0.34 |
| PNUE | -0.15 | -0.14 | -0.18 | 0.28 | 0 | -0.33 | -0.04 | 0.2 | -0.26 | -0.1 | -0.08 | -0.15 | 0.01 | -0.17 | **0.46** | **0.57** | **0.86** |  | **0.89** | 0.1 | -0.12 | **-0.54** | 0.16 | -0.39 | 0.3 |
| PPUE | -0.22 | -0.19 | -0.2 | 0.21 | -0.2 | **-0.41** | -0.15 | -0.05 | -0.14 | -0.08 | -0.39 | -0.21 | **0.47** | -0.05 | 0.15 | 0.33 | **0.77** | **0.89** |  | 0.27 | -0.08 | **-0.58** | 0 | -0.33 | 0.39 |
| WUE_i_ | -0.27 | -0.22 | -0.24 | -0.33 | -0.25 | -0.13 | -0.24 | -0.16 | 0.19 | 0.06 | **-0.39** | 0.02 | **0.63** | 0.02 | **-0.82** | **-0.51** | -0.14 | -0.19 | 0.13 |  | 0.26 | 0.11 | 0.05 | -0.39 | -0.03 |
| *D*_vein_ | -0.28 | -0.09 | -0.3 | **0.45** | **-0.46** | 0.38 | 0.11 | -0.32 | 0.12 | 0.02 | 0.08 | 0.08 | -0.1 | 0.04 | 0.09 | 0.09 | -0.05 | -0.06 | -0.1 | -0.07 |  | **0.44** | -0.06 | 0.26 | -0.22 |
| WD | 0 | 0.16 | 0.01 | 0.16 | -0.25 | **0.43** | **0.41** | **-0.46** | **0.59** | -0.22 | -0.2 | -0.39 | 0.05 | -0.11 | -0.21 | -0.31 | **-0.62** | **-0.55** | **-0.46** | 0.03 | **0.48** |  | -0.18 | **0.48** | **-0.66** |
| *D*_h_ | -0.03 | -0.21 | 0 | -0.16 | 0.26 | 0.06 | -0.05 | **0.57** | -0.29 | 0.03 | 0.18 | 0.24 | -0.23 | -0.3 | 0.01 | 0.17 | 0.11 | 0.11 | -0.01 | 0.23 | -0.35 | **-0.41** |  | **-0.57** | **0.55** |
| VD | 0.12 | 0.2 | 0.13 | **0.45** | -0.1 | 0.31 | 0.29 | **-0.41** | 0.34 | -0.15 | -0.04 | -0.26 | -0.11 | 0.17 | 0.31 | 0.04 | -0.3 | -0.24 | -0.27 | **-0.58** | 0.37 | **0.55** | **-0.72** |  | -0.37 |
| *K*_t_ | -0.24 | -0.28 | -0.26 | -0.23 | 0.16 | -0.07 | **-0.54** | **0.4** | **-0.44** | 0.29 | 0.19 | 0.36 | 0.04 | 0.11 | 0 | 0.16 | 0.36 | 0.23 | 0.22 | 0.22 | **-0.44** | **-0.76** | **0.56** | **-0.61** |  |

Note: See Table 2 for trait abbreviations. Green font: *p* **<** 0.1; bold font: *p* **<** 0.05; red font: *p* **<** 0.01.The unit of δ^13^C is (–‰).

**Table S4.** Tests of the SMA regression slopes, intercepts, and shifts along the common slopes for log-log bivariate relationships between two habitats.

| y ~ x | Common slope/slope | | |  | Intercept | | |  | Shifts along the common slope |
| --- | --- | --- | --- | --- | --- | --- | --- | --- | --- |
|  | Rainforest | Savanna | *p* |  | Rainforest | Savanna | *p* |  | *p* |
| log *A*_a_ ~ log *g*_s_ | 0.70 | | 0.446 |  | 1.41 | 1.56 | **<0.001** |  | 0.167 |
| log *A*_a_ ~ log SD | 0.79 | | 0.138 |  | -0.91 | -0.85 | 0.309 |  | 0.540 |
| log *g*_s_~ log SD | 0.77 | 1.43 | **0.039** |  |  |  |  |  |  |
| log WUE_i_ ~ log *g*_s_ | -0.55 | | 0.132 |  | 1.28 | 1.38 | **0.007** |  | **<0.001** |
| log GCL ~ log SD | -0.436 | | 0.846 |  | 2.40 | 2.40 | 0.937 |  | 0.246 |
| log GCL ~ log *D*_vein_ | -0.494 | | 0.690 |  | 1.73 | 1.77 | 0.290 |  | 0.494 |
| log VD ~ log *D*_h_ | -1.20 | | 0.623 |  | 3.56 | 3.78 | **0.008** |  | **<0.001** |
| log *K*_t_~ log *D*_h_ | 3.16 | 1.79 | **0.013** |  |  |  |  |  |  |
| log *K*_t_ ~ log WD | -7.98 | -3.29 | **0.001** |  |  |  |  |  |  |

If the slopes were not significantly different between rainforest and savanna liana species, then the common slopes were given; if no significant differences in slopes and shifts in intercepts were detected, shifts along the common slopes were then tested for.

Significant differences (*p* **<** 0.05) are indicated in bold. See Table 2 for trait abbreviations.

**Table S5.** Phylogenetic signals for each trait measured.

| Combined | | |  | Savanna | |  | Rainforest | |
| --- | --- | --- | --- | --- | --- | --- | --- | --- |
|  | K | PIC.variance.P |  | K | PIC.variance.P |  | K | PIC.variance.P |
| LT | 0.351 | **0.036** |  | 0.567 | 0.103 |  | 0.830 | **0.011** |
| PT | 0.218 | 0.579 |  | 0.432 | 0.265 |  | 0.453 | 0.177 |
| ST | 0.329 | **0.040** |  | 0.684 | **0.042** |  | 0.436 | 0.136 |
| SD | 0.438 | **0.010** |  | 0.307 | 0.887 |  | 0.936 | **0.004** |
| GCL | 0.638 | **0.002** |  | 0.457 | 0.185 |  | 1.081 | **0.003** |
| LD | 0.251 | 0.321 |  | 0.260 | 0.810 |  | 0.575 | **0.046** |
| LMA | 0.169 | 0.883 |  | 0.505 | 0.107 |  | 0.238 | 0.926 |
| LS | 0.549 | **0.003** |  | 0.289 | 0.703 |  | 0.588 | **0.021** |
| LDMC | 0.353 | **0.011** |  | 0.537 | 0.074 |  | 0.592 | **0.041** |
| N | 0.303 | 0.064 |  | 0.409 | 0.385 |  | 0.374 | 0.308 |
| P | 0.270 | 0.194 |  | 0.328 | 0.678 |  | 0.427 | 0.222 |
| K | 0.439 | **0.007** |  | 0.476 | 0.191 |  | 0.778 | **0.014** |
| N/P | 0.349 | **0.028** |  | 0.452 | 0.205 |  | 0.567 | **0.043** |
| δ^13^C | 0.232 | 0.699 |  | 0.388 | 0.320 |  | 0.375 | 0.383 |
| *g*_s_ | 0.264 | 0.178 |  | 0.951 | **0.002** |  | 0.363 | 0.314 |
| *A*_a_ | 0.348 | 0.197 |  | 0.415 | 0.445 |  | 0.571 | 0.210 |
| *A*_m_ | 0.270 | 0.473 |  | 0.434 | 0.272 |  | 0.314 | 0.880 |
| PNUE | 0.300 | 0.356 |  | 0.475 | 0.182 |  | 0.319 | 0.818 |
| PPUE | 0.229 | 0.600 |  | 0.358 | 0.387 |  | 0.291 | 0.773 |
| WUE_i_ | 0.201 | 0.632 |  | 1.262 | **0.002** |  | 0.684 | **0.032** |
| *D*_vein_ | 0.420 | **0.009** |  | 0.546 | 0.138 |  | 0.684 | **0.012** |
| WD | 0.317 | 0.166 |  | 0.434 | 0.249 |  | 0.513 | 0.102 |
| *D*_h_ | 0.380 | 0.090 |  | 0.713 | 0.069 |  | 0.312 | 0.486 |
| VD | 0.266 | 0.378 |  | 0.410 | 0.365 |  | 0.356 | 0.323 |
| *K*_t_ | 0.718 | 0.073 |  | 0.476 | 0.162 |  | 0.386 | 0.334 |

Note: See Table 2 for trait abbreviations.

**Table S6.** Loadings of 25 functional traits on the first four principal components for 36 liana species from tropical seasonal rainforest and savanna habitats.

| Traits | PC1 | PC2 | PC3 | PC4 |
| --- | --- | --- | --- | --- |
| LT | 0.101 | -0.332 | -0.322 | 0.067 |
| PT | 0.030 | -0.214 | -0.381 | 0.075 |
| ST | 0.069 | -0.313 | -0.267 | -0.017 |
| SD | -0.060 | 0.332 | -0.264 | -0.117 |
| GCL | 0.053 | -0.208 | 0.047 | 0.145 |
| LD | -0.189 | 0.217 | -0.003 | -0.256 |
| LMA | -0.162 | -0.071 | -0.330 | -0.126 |
| LS | 0.263 | 0.089 | -0.069 | -0.163 |
| LDMC | -0.355 | 0.052 | 0.049 | -0.078 |
| N | 0.232 | 0.146 | 0.140 | -0.093 |
| P | 0.304 | 0.049 | -0.136 | -0.083 |
| K | 0.261 | -0.048 | 0.045 | -0.070 |
| N/P | -0.228 | 0.055 | 0.308 | 0.038 |
| δ^13^C | 0.204 | 0.081 | 0.145 | -0.302 |
| *g*_s_ | 0.165 | 0.295 | -0.291 | -0.045 |
| *A*_a_ | 0.102 | 0.264 | -0.321 | 0.209 |
| *A*_m_ | 0.169 | 0.281 | -0.016 | 0.363 |
| PNUE | 0.046 | 0.223 | -0.103 | 0.457 |
| PPUE | -0.101 | 0.225 | 0.102 | 0.418 |
| WUE_i_ | -0.154 | -0.194 | 0.126 | 0.269 |
| *D*_vein_ | -0.120 | 0.335 | -0.070 | -0.224 |
| WD | -0.276 | 0.001 | -0.150 | -0.138 |
| *D*_h_ | 0.282 | -0.038 | 0.119 | -0.061 |
| VD | -0.256 | -0.032 | -0.196 | 0.109 |
| *K*_t_ | 0.274 | -0.074 | 0.175 | 0.100 |
| Proportion of variance explained (%) | 26.68 | 17.99 | 12.68 | 12.39 |

Note: See Table 2 for trait abbreviations. The unit of δ^13^C is (–‰).

**Table S7.** Means ± SE of 25 functional traits for evergreen and deciduous liana species.

| **Traits** | **Evergreen (*n* = 16)** | **CV** | **Deciduous (*n* = 20)** | **CV** | ***t*** | ***p*** |
| --- | --- | --- | --- | --- | --- | --- |
| LT | 217.81 ± 21.94 | 0.4 | 196.6 ± 16.39 | 0.37 | -1.72 | 0.097 |
| PT | 76.75 ± 7.97 | 0.42 | 62.7 ± 4.02 | 0.29 | -1.09 | 0.282 |
| ST | 103.94 ± 12.9 | 0.5 | 82.65 ± 7.39 | 0.4 | -2.00 | 0.054 |
| SD | 293.31 ± 35.39 | 0.48 | 279.7 ± 27.02 | 0.43 | 0.40 | 0.689 |
| GCL | 22.88 ± 1.21 | 0.21 | 22.93 ± 1.17 | 0.23 | 0.55 | 0.587 |
| LD | 297.62 ± 29.02 | 0.39 | 340.3 ± 34.97 | 0.46 | 0.52 | 0.609 |
| LMA | 63.12 ± 6.55 | 0.41 | 56.5 ± 4.45 | 0.35 | -2.24 | **0.032** |
| LS | 65.78 ± 11.51 | 0.7 | 40.84 ± 8.74 | 0.96 | -1.60 | 0.118 |
| LDMC | 0.27 ± 0.03 | 0.37 | 0.31 ± 0.02 | 0.26 | 1.32 | 0.199 |
| N | 25.01 ± 1.65 | 0.26 | 23.68 ± 1.35 | 0.26 | -0.12 | 0.902 |
| P | 1.82 ± 0.21 | 0.47 | 1.58 ± 0.14 | 0.4 | -0.48 | 0.633 |
| K | 11.87 ± 1.74 | 0.59 | 13.06 ± 1.77 | 0.61 | 0.07 | 0.947 |
| N/P | 15.51 ± 1.12 | 0.29 | 16.35 ± 1.13 | 0.31 | 0.57 | 0.574 |
| δ^13^C | 29.76 ± 0.54 | 0.07 | 28.05 ± 0.31 | 0.05 | -2.59 | **0.016** |
| *g*_s_ | 0.3 ± 0.04 | 0.54 | 0.23 ± 0.02 | 0.4 | 0.00 | 0.999 |
| *A*_a_ | 10.82 ± 0.85 | 0.31 | 12.06 ± 0.84 | 0.31 | 1.46 | 0.154 |
| *A*_m_ | 205.62 ± 25.24 | 0.49 | 225 ± 24.13 | 0.48 | 2.28 | **0.029** |
| PNUE | 111.94 ± 9.44 | 0.34 | 139.15 ± 16.05 | 0.52 | 2.76 | **0.009** |
| PPUE | 3.68 ± 0.31 | 0.34 | 5.08 ± 0.7 | 0.62 | 2.68 | **0.011** |
| WUE_i_ | 45.25 ± 6.1 | 0.54 | 58.95 ± 3.96 | 0.3 | 1.42 | 0.164 |
| *D*_vein_ | 7.16 ± 0.71 | 0.39 | 7.07 ± 0.67 | 0.42 | 1.18 | 0.247 |
| WD | 0.45 ± 0.03 | 0.28 | 0.5 ± 0.03 | 0.23 | 0.77 | 0.444 |
| *D*_h_ | 113.19 ± 17.29 | 0.61 | 67.25 ± 8.16 | 0.54 | -3.30 | **0.002** |
| VD | 23.88 ± 8.91 | 1.49 | 53.65 ± 10.04 | 0.84 | 3.74 | **0.001** |
| *K*_t_ | 173.3 ± 89.17 | 2.06 | 57.08 ± 18.62 | 1.46 | -1.70 | 0.099 |

Note: The *t* and *p* values of independent-samples *t* test were given. See Table 2 for trait abbreviations.


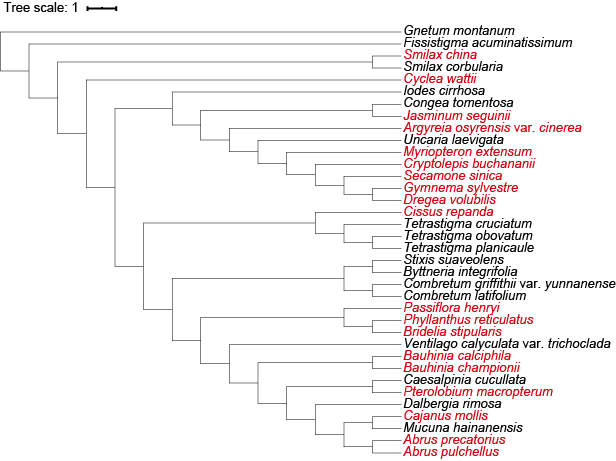


**Figure S1.** Phylogenetic tree of the 36 liana species from savanna and tropical seasonal rainforest habitats. The red ones are from the savanna and the black ones are from the tropical seasonal rainforest.


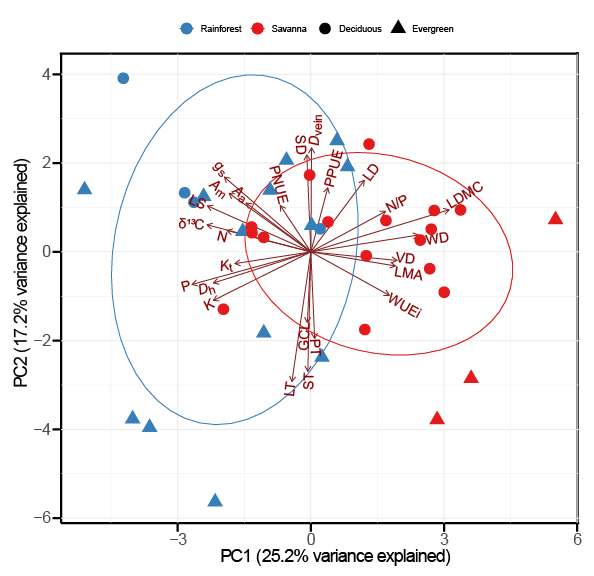


**Figure S2.** The biplot of first two axes of the phylogenetic principal component analysis for the 25 leaf and stem functional traits and the loadings of the 36 liana species from savanna (red) and tropical seasonal rainforest (blue). See Table 2 for trait abbreviations. All variables were log_10_-transfored before analysis. The unit of δ^13^C is (–‰).
